# Chromatographic capture of cells to achieve single stage clarification in recombinant protein purification

**DOI:** 10.1002/btpr.3227

**Published:** 2021-12-08

**Authors:** Aaron Almeida, David Chau, Thomas Coolidge, Hani El‐Sabbahy, Steven Hager, Kevin Jose, Masa Nakamura, Alexei Voloshin

**Affiliations:** ^1^ Manufacturing Process Optimization Catalent Biologics Madison Wisconsin USA; ^2^ Separation and Purification Sciences Division 3M Company Saint Paul Minnesota USA

**Keywords:** chromatographic clarification, clarification, DNA reduction, downstream processing, Protein A chromatography

## Abstract

Recent advancements in cell culture engineering have allowed drug manufacturers to achieve higher productivity by driving higher product titers through cell line engineering and high‐cell densities. However, these advancements have shifted the burden to clarification and downstream processing where the difficulties now revolve around removing higher levels of process‐ and product‐related impurities. As a result, a lot of research efforts have turned to developing new approaches and technologies or process optimization to still deliver high quality biological products while controlling cost of goods. Here, we explored the impact of a novel single use technology employing chromatographic principle‐based clarification for a process‐intensified cell line technology. In this study, a 16% economic benefit ($/g) was observed using a single‐use chromatographic clarification compared to traditional single‐use clarification technology by improving the overall product cost through decreased operational complexity, higher loading capacity, increased product recovery, and higher impurity clearance. In the end, the described novel chromatographic approach significantly simplified and enhanced the cell culture fluid harvest unit operation by combining the reduction of insoluble and key soluble contaminants of the harvest fluid into a single stage.

## 1 INTRODUCTION

Monoclonal antibody (mAb) therapeutics is one of the biggest product segments in the pharmaceutical industry. Six out of the top 10 pharmaceuticals sold in 2018 were mAbs and a staggering 18 antibodies were approved the following year by the US FDA.^1^ The global mAb market has been valued at USD $143.5 billion and expected to grow at a compound annual growth rate of 15% from 2020 to 2026. To meet the market demand, there has been an increased expenditure in research and development in the industry to accelerate the speed from discovery to clinic. In the last few years, process simplification and process intensification have emerged as key areas of focus when designing and implementing bioprocessing strategies for promoting a more efficient mAb production process. Thus, advancements in novel technologies that promote these areas will play a major role in supporting the continued growth of the mAb therapeutic market space.

In the past couple of decades, the deployment of platform technologies such as using Chinese hamster ovary (CHO) cells as the expression platform have emerged as a standard practice in the industry to streamline the manufacturing process for higher efficiencies and flexibility. In the last 5 years, over 80% of approved recombinant therapeutic mAbs were expressed in mammalian cell culture systems and predominately in CHO cell lines.^2^, ^3^ Further advancements in the understanding of cell biology and cell culture have led to higher cell densities (>25 × 10^6^ cells/ml), increased cell productivity and longer cell culture durations which result in a higher titer and ultimately higher productivity. However, the increased cell mass and corresponding increase in soluble and insoluble contaminant loading have challenged the traditional approaches for separation and purification operations shifting the burden further downstream.

Traditionally, the cell culture harvest unit operation is the first step for clarification and responsible for the removal of cells, cell debris, and other large insoluble aggregates. Legacy clarification strategies use a combination of centrifugation, depth filtration, and membrane filtration for clarifying cell culture fluid (CCCF). Together, these approaches are still widely regarded as the benchmark for clarification performance. Centrifugation is a popular approach using centrifugal force to separate large components in a mixture according to their density and particle size properties.^4^, ^5^ Depth filtration relies on a complex porous media containing a mixture of naturally derived filter aids, cellulose, and a resin binder to retain large particulates while letting the soluble product (e.g., mAb) through based on a size exclusion principle. Depth filtration have been the most prevalent single‐use technology deployed for clarification in many biopharmaceutical settings. Additionally, novel filter media designs with a wider range of pore sizes and different filter media morphology have been explored to improve filter media utilization.^6^ However, the fundamental principle for separation has largely been limited to using size and density as the basic principle of separation. In recent years, newer concepts such as acoustic wave capture, precipitation, and flocculation, have been explored to replace and/or complement legacy clarification strategies to overcome capacity limitations, but still primarily rely on traditional size and sedimentation clarification principles. As a result, each of the legacy and emerging clarification technologies have encountered similar technical and economic challenges that have been described more extensively in other reviews and articles.^7^, ^8^, ^9^

In this study, we explored a novel anion exchange (AEX) chromatographic approach designed for cell culture harvest in a single use format. The adoption and innovation in single use technology have proven to further the transformation of the biomanufacturing industry to be more efficient and flexible from traditional stainless‐steel technology. Single use technology can be used and disposed of without having the drawbacks of capital investments and cost associated with cleaning and validation along with shorter turnaround time which would all ultimately lead to higher productivity.^10^, ^11^ However, as bioprocesses have evolved to yield higher cell densities, the ability to clarify current and future cell cultures with higher levels of insoluble and soluble impurities with traditional single use clarification technologies have proven to be difficult. Current single use technology for cell culture harvest are primarily constructed out of fibers from naturally derived ingredients to provide the structure for mechanical sieving, and recent studies have reported the incorporation of adsorptive properties to aid for a better separation.^12^, ^13^ Khanal et al. specifically goes into the effect of using highly charged resin binders combined with a porous depth filter media to enhance the adsorption of soluble contaminants.^12^ Thus, charge‐based separation that was typically reserved for downstream processing are now being explored in the clarification stages of cell culture harvest. The objective of this study was to evaluate the potential of using a novel single use technology resembling chromatography for the clarification of raw CCF referred to as 3M™ Harvest RC Chromatographic clarifier in this study. This single stage would utilize fibrous AEX technology engineered to capture the entire range of particles found in raw cell culture broth from cells to DNA in a single‐step approach for clarification.

Current chromatography technologies for mAb production exist in the form of columns, resins, or functional membranes. Chromatography generally exploits the physical and chemical differences between biomolecules to achieve a higher degree of separation compared to upstream filtration. However, the increase in cell density, cellular debris and other impurities have now caused new challenges for both clarification and downstream chromatography. The primary limitation of all the current chromatographic media types is that they are not well suited to handle the initial level of large particulates present in cell culture. Large quantities of solids can easily foul chromatographic devices that will reduce overall performance and effectiveness.^14^ In the last decade, we saw the attempt of using chromatography for clarification in the form of expanded bed adsorption (EBA). However, the original concept of EBA was to use chromatography to not only achieve high separation efficiency but also capture the product.^15^, ^16^ In certain applications, EBA had success where the levels of contaminants were low and the titer was moderate enough to where the EBA could be deployed to replace several traditional unit operations, namely centrifugation, filtration, and capture chromatography. However, like other column and bead‐based technology, EBA was susceptible to fouling and clogging once the impurity loading specifically with cells and cell debris got too high. There were other drawbacks associated with deploying EBA at manufacturing scale which involved the complexity to clean and validate the EBA column that needed to be regenerated and re‐equilibrated before each use.^15^ In addition, the flow rate of the column can be very restricted in order to keep the bead‐based bed suspended thus slowing down the process.^17^ Thus, chromatographic separation has been primarily reserved for small soluble particles in downstream processing.

More recently, there have been attempts to use a different method of chromatography in a single use AEX chromatographic media to effectively remove soluble impurities such as host cell proteins (HCP), DNA, viruses, and soluble aggregates during the raw cell culture harvest.^18^, ^19^, ^20^ Castro‐Forero et al. have shown the ability to use a chromatographic media during the clarification to remove high levels of soluble impurities such as DNA and HCPs. The high AEX capacity showed the capability to reduce up to 99.99% DNA and 24% HCPs with chromatographic clarification compared to conventional clarification strategy alleviating the burden on downstream purification.^21^ We have previously reported that this approach can be effectively used across multiple molecules and is platformable.^22^ It has been further reported that the advantages of deploying chromatographic clarification in this manner have led to improved viral clearance performance and reduced impurity challenges to downstream polishing columns and filters by generating a cleaner filtrate prior to the capture step.^23^, ^24^ Similar to conventional chromatographic modalities, current chromatographic clarification can only be achieved by combining adsorptive hybrid filters with other conventional clarification techniques to remove larger insoluble contaminants.

**FIGURE 1:**
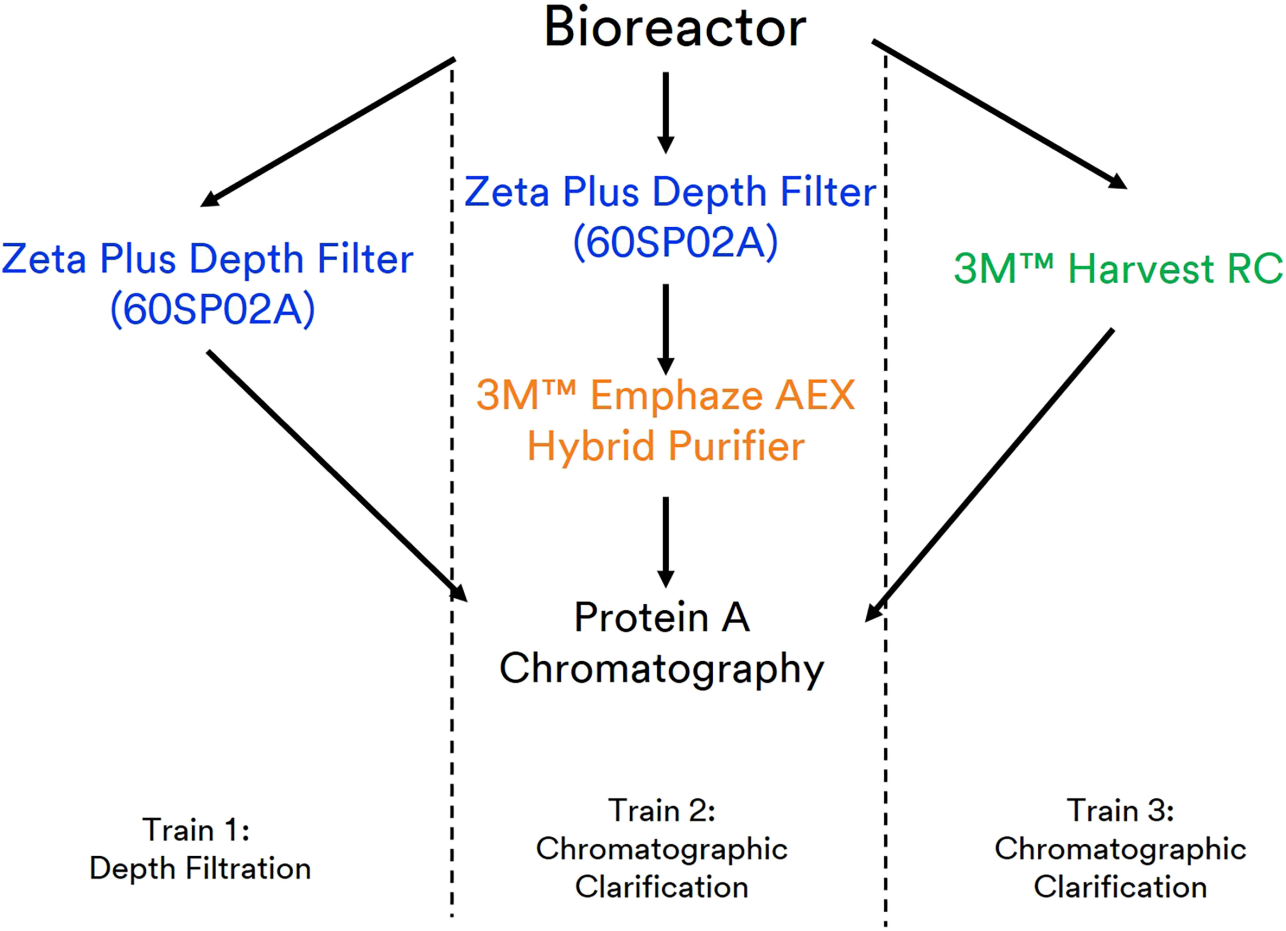
Process flow diagram of different single use clarification trains combined with Protein A

To our knowledge, this is the first reported study to utilize chromatography during clarification without any additional conventional clarification techniques needed. In this study, we explored the impact and savings that can be achieved by comparing multiple single use technology for clarification to understand the impact of using a novel single use technology to achieve chromatographic clarification (Figure 1).This article primarily addresses the use of a single stage fiber chromatographic clarification technology for high cell density cultures as compared to traditional clarification techniques. The GPEx® and GPEx® Boost cell lines studied within this article were chosen as good clarification challenge cases, representative of process intensified mAb production conditions expected for modern pharmaceutical production processes.^25^ Clarification is a critical unit operation in the production of mAbs because it directly affects yield, product consistency and performance of downstream unit operations. By implementing the novel strategies presented in this study, we have shown the ability to demonstrate process intensification and compression to drive improved yields and lower operating costs.

## 2 MATERIALS AND METHODS

### 2.1 Cell culture

Three proprietary CHO cell lines were used to produce harvests contaminant recombinant monoclonal antibodies (mAb 1, mAb 2, and mAb 3). Two Catalent monoclonal GPEx CHO cell line expressing an IgG antibody and one Catalent monoclonal GPEx Boost CHO cell line expressing an Fc‐fusion protein were used for this study. These three cell lines were grown in commercially available cell culture medium and passaged every 3–4 days in shaker flasks in a cell incubator until a suitable cell number was attained to inoculate the production vessel.

The production phase was performed in single‐use bioreactors incorporating a fed‐batch process. The basal medium used was a commercially available cell culture medium supplemented with matching commercially available concentrated feeds at regular intervals to maintain a healthy cell culture environment. The production vessel was monitored daily, and process parameters maintained within the platform limits.

### 2.2 Cell culture conditions

The cultures were harvested when protein production leveled off, reaching a protein concentration of 1.00 g/L for the 3 M cell line, 1.86 g/L for the GPEx cell line, and 6.2 g/L for the GPEx Boost line. The detailed information for each cell culture used in this study can be found in Table 1 below.

**TABLE 1:** Different mAb cell culture characteristic

Target modality	Host organism	Modality	Product pI	Cell density (10^6^/ml)	Harvest viability (%)	Turbidity (NTU)	Titer (g/L)	Pack cell volume (%)
mAb 1	CHO	IgG	~7.95	9.15	70	1439	1.0	3.7
mAb 2	CHO	IgG	7.95	14	75	2597	1.86	4.2
mAb 3	CHO	Fc‐fusion	8.20	20	97.6	>3000	6.2	7.4

### 2.3 Clarification

The 3M™ Zeta Plus™ 60SP02A depth filters were first prepared by flushing with ~54 L/m^2^ of Water for Injection and then equilibrated with 0.05 M Tris, 0.150 M NaCl, and pH 7.5 solution. The 3M™ Emphaze™ AEX Hybrid Purifier chromatographic clarifiers were prepared separately by flushing >54 L/m^2^ of 0.05 M Tris, 0.150 M NaCl, and pH 7.5 solution prior to being placed in line as a secondary stage to the 60SP02A. The 3M™ Harvest RC clarifiers were prepared by equilibrating with >15 L/m^2^ of 0.05 M Tris, 0.050 M NaCl, and pH 7.5 solution. The 3M™ Zeta Plus™ 60SP02A filter trains were operated at 150 L per meter square per hour (LMH) whereas Harvest filters were operated at 200 LMH. A terminal 0.2 μm polyethersulfone (PES) filter was used for each filter train.

### 2.4 Protein A purification chromatography

Capture chromatography was performed on an AKTA™ avant 150 using Cytiva's Mabselect™ PrismA resin packed in a XK50/30 column. The purifications utilized a 4‐minute residence time while targeting an approximately 40 g/L load of product. Tris/Acetate buffering systems were implemented including high salt and intermediate pH wash buffers. Collection criteria of the eluate was 50 mAU upslope and downslope (2 mm pathlength). Collected eluate was virally inactivated for 30 min at pH 3.5 using 2 M acetic acid. Virally inactivated eluate was neutralized to approximately pH 7.0 using 2 M Tris base prior to final filtration using a 0.2 μm PES filter.

### 2.5 Host cell DNA quantification

Detection of CHO DNA by quantitative polymerase chain reaction (qPCR) was carried out using the resDNASEQ preparation kit from Thermo Fischer as part of an integrative system that includes sample preparation, TaqMan® assay and master mix, standard DNA, instruments, and software (AccuSEQ™) and has a limit of quantitation of 6 pg DNA/ml. Results are generated from a 7500FEST real‐time PCR system and reported as pg DNA/mg of protein.

### 2.6 HCP quantification

Detection of HCP by enzyme‐linked immunoassay (ELISA) using Cygnus CHO HCL ELISA Kit F550‐1 kit. The antibodies used in this procedure are polyclonal and designed to broadly react with most HCP's that might co‐purify with the desired product.

### 2.7 Protein quantification

Protein quantification in media was carried out using a 0.1 ml (2.1 × 30 mm) POROS A 20 μm column with a six point internal calibration standard in sample matrix using a bind/elute buffer system. A standard curve was generated off the area of the peaks, and sample areas were evaluated against the standard curve.

### 2.8 Turbidity and particle size analysis

Turbidity of the CCCF was measured using an ORION™ AQ4500 turbidity meter (Thermo Fisher Scientific, Waltham, MA) in NTU units. Dynamic light scattering (DLS) particle size distribution in CCCF was measured using a Nanotrac Flex DLS Analyzer (Microtrac, Montgomeryville, PA). Particle size distribution from 1 to 6 μm was measured in intensity mode.

### 2.9 BioSolve modeling

Cost of goods (CoGs) modeling was conducted using BioSolve Version 8.1 (Biopharm Services, Chesham UK), a Microsoft Excel based model that employs an extensive database of costs including equipment, materials and consumables drawn from the biopharmaceutical industry. The facility and process assumptions used for the Biosolve model are set out in Table 2.

**TABLE 2:** Facility and process parameters

Bioreactor size	2000 L
Bioreactor number	6
Number of reactors harvested together	1
Facility output	100 batches per year
mAb titer	1.86 g/L

In order to understand the impact of 3M™ Harvest RC on the manufacturing costs for a mAb product, two main processes were modeled: a state of the art baseline process, which contained a two‐stage clarification train including a primary depth filter followed by chromatographic clarification using the 3M™ Emphaze™ AEX Hybrid Purifier; and a next generation process with a single clarification step, 3M™ Harvest RC solution. All other unit operations were kept the same between the two processes. The key parameters for each of the unit operations including loading, unit operation size and yield are shown in Table 3.

**TABLE 3:** Details of the downstream unit operations modeled in each of the processes including loading, size, and yields

Process	Parameter	Clarification		Sterile membrane	Protein A chromatography
Baseline	Size	Zeta Plus Depth Filter	Emphaze AEX Hybrid Purifier	5 capsules	57 L
		15 capsules	9 capsules		
	Load	88 L/m^2^	176 L/m^2^	400 L/m^2^	35 g/L
	Yield	90%	98%	98%	90%
Hypothetical ‐yield effect only	Size	Zeta Plus Depth Filter	Emphaze AEX Hybrid Purifier	5 capsules	57 L
		15 capsules	9 capsules		
	Load	88 L/m^2^	176 L/m^2^	400 L/m^2^	35 g/L
	Yield	100%	98%	98%	90%
Hypothetical single step clarification	Size	Hypothetical single step clarifier		5 capsules	57 L
		24 capsules			
	Load	54 L/m^2^		400 L/m^2^	35 g/L
	Yield	98%		98%	90%
Harvest RC	Size	3M™ Harvest RC		4 capsules	57 L
		10 capsules			
	Load	132 L/m^2^		400 L/m^2^	35 g/L
	Yield	98%		98%	90%

To understand the impact of particular features for a single stage clarification stage using 3M™ Harvest RC, incremental changes were made to the baseline process to understand the impact 3M™ Harvest RC has on mAb manufacturing costs. To understand where the cost savings could be realized deploying this new technology, two hypothetical situations showing the impact of having higher product recovery and condensing of multiple stages to one were modeled using Biosolve according to Table 3 to understand the impact of the these two features that were realized when deploying 3M™ Harvest RC. The key parameters for each of the unit operations in these hypothetical processes including loading, unit operation size and yield are also shown in Table 3. In the first hypothetical process, the yield for the primary clarification depth filter step was set at 100% so that the overall clarification stages would mimic the 98% overall yield from the 3M™ Harvest RC. This would help us understand the impact of having a higher yield. The second hypothetical process was modeled by combining two clarification stages from the baseline process into a single hypothetical step. This single hypothetical clarification step was modeled such that it had no overall impact on the number or cost of consumables or hardware used in clarification.

## 3 RESULTS AND DISCUSSION

### 3.1 Chromatographic clarification with 3M™ Harvest RC

**FIGURE 2:**
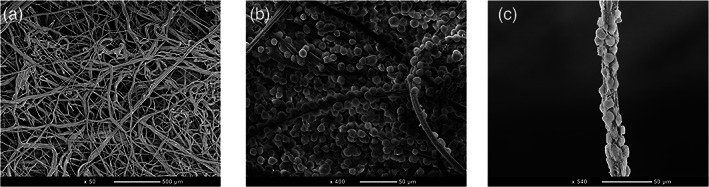
Scanning electron microscopy image of 3M™ Harvest RC. (a) Functionalized polypropylene fibers in the nonwoven bed. (b) Cells attached to the nonwoven bed of the 3M™ harvest RC. (c) Cells attached to an individual functionalized polypropylene Fiber of the 3M™ Harvest RC

In this study, we explored the concept of chromatographic clarification using a fibrous AEX media referred to as 3M™ Harvest RC. Current approaches for chromatographic clarification require multiple stages for the removal of large particles followed by smaller and soluble impurities. This study evaluated the possibility of using 3M™ Harvest RC as a stand‐alone stage for chromatographic clarification. As shown in Figure 2a, the 3M™ Harvest RC technology is a bed of quaternary ammonium functionalized polypropylene fibers. Using scanning electron microscopy (SEM), cells were found adsorbed and captured throughout the matrix of the functional nonwoven bed (Figure 2b). Cells, cell debris, and other negatively charged soluble contaminants were observed to be bound to individual functionalized fibers suggesting the ability to use chromatographic means for cell capture compared to traditional size or density approaches (Figure 2c).

**FIGURE 3:**
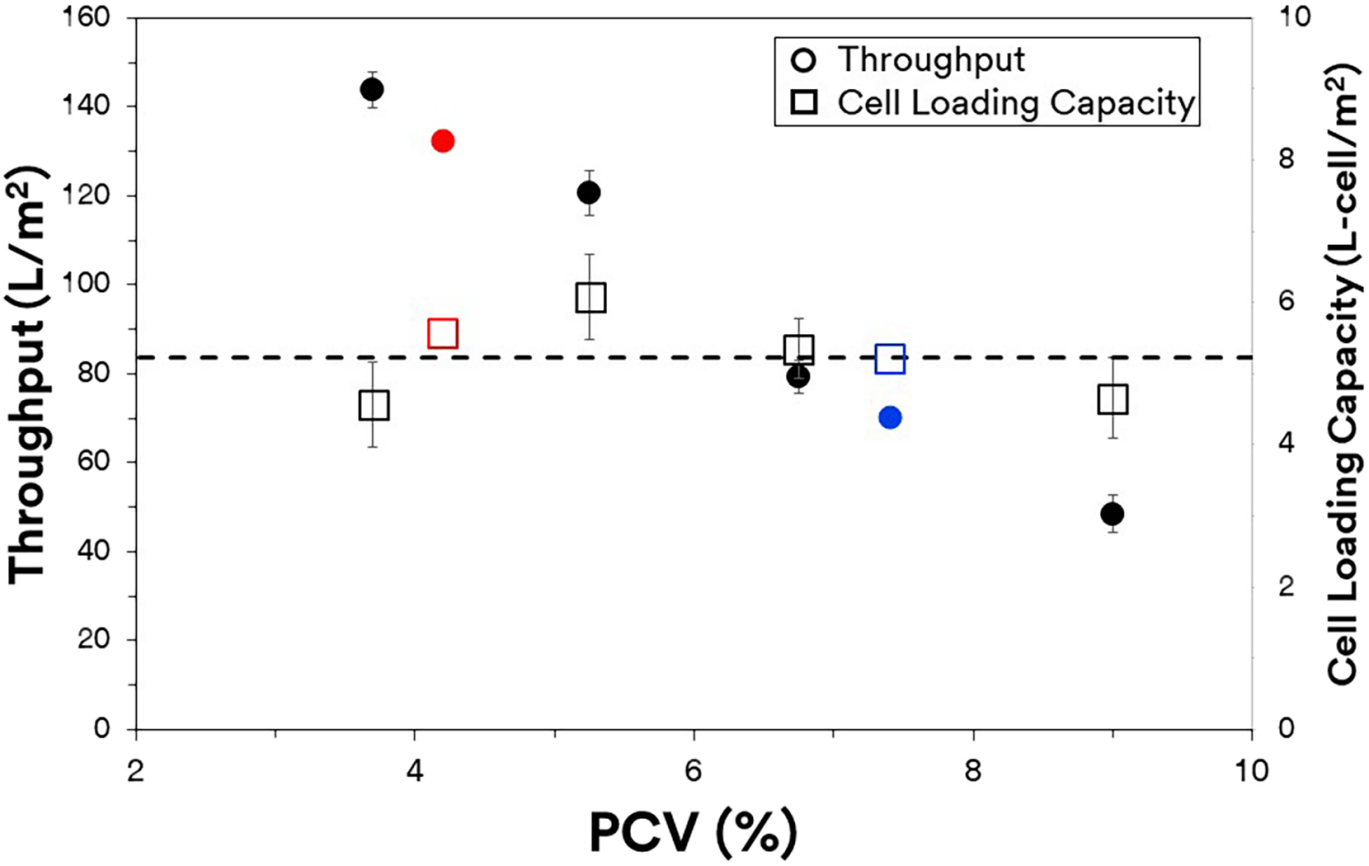
Effect of pack cell volume versus throughput for the 3M™ harvest RC. mAb 1 (black) mAb 2 (red) mAb 3 (blue). Dashed line—average of cell loading capacity for different cell cultures

The robustness and consistency to clarify different cell cultures with varying packed cell volume (PCV) or solid content was evaluated using the 3M™ Harvest RC technology (Table 1). A throughput of ~143.8 L/m^2^ was achieved for the first cell culture corresponding to a 3.7% PCV cell culture with a cell density of 9.15 × 10^6^ cells/ml. The solid content was further concentrated to 5.25%, 6.75%, and 9% PCV to observe the scalability between throughput and solid content. It was observed that there was a direct correlation between the two parameters (Figure 3). Two additional cell cultures (mAb 2 and 3) more representative of modern challenging cell densities of 14 and 20 ( × 10^6^ cells/ml) corresponding to 4.2 and 7.4% PCV, were also challenged onto the 3M™ Harvest RC technology. These additional cell cultures were chosen to represent more modern and challenging cell cultures and modalities that typical clarification strategies would struggle with. Despite the higher cell densities and solid loading, a throughput of 132 and 70 L/m^2^ was achieved, respectively (Figure 3). The throughputs of the 3M™ Harvest RC technology was found to decrease accordingly with higher cell density cultures but in a predictable and scalable manner. This suggests a uniform and consistent utilization of the chromatographic media regardless of the cell culture challenge. To confirm this, the loading capacity for each of the challenge conditions was calculated by dividing the amount of solid content loaded onto 3M™ Harvest RC by the filter media area. This was confirmed to be ~5.25 ± 0.52 L of cells/m^2^ of 3M™ Harvest RC media. A linear relationship was observed between the PCV and throughput indicating that the higher solid content did not necessarily contribute to any filter caking that is typically observed with traditional clarification strategies using depth or membrane filtration.^26^, ^27^

**FIGURE 4:**
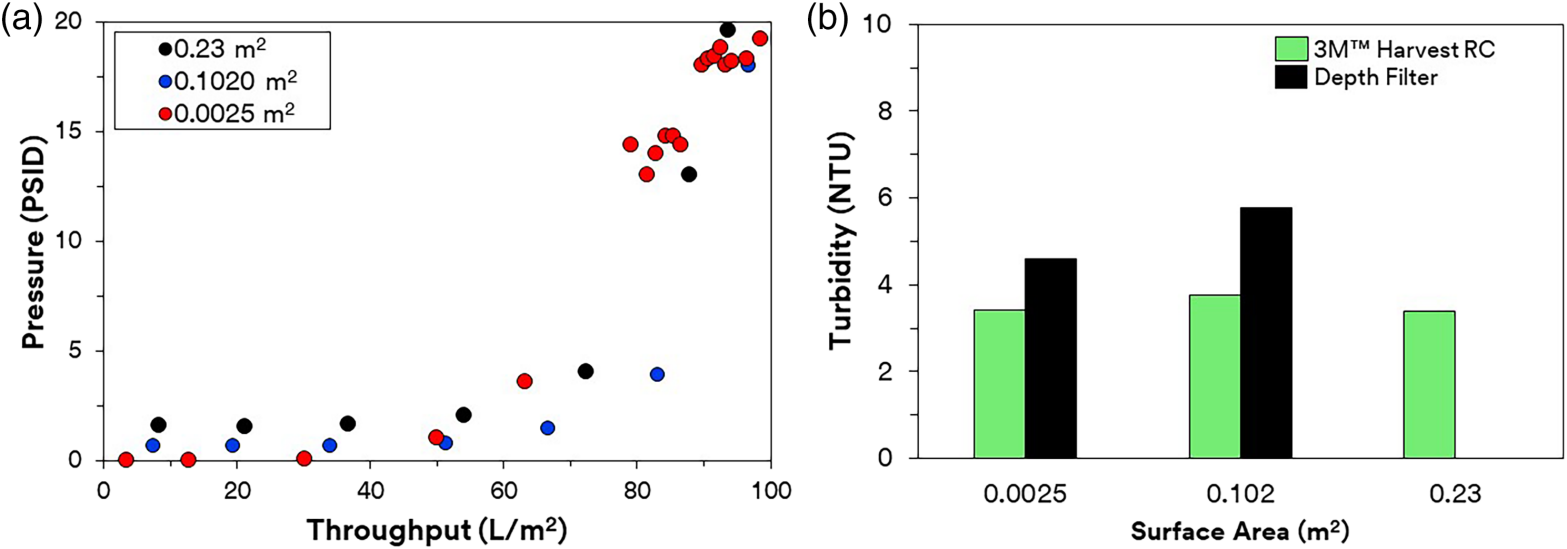
Performance of 3M™ Harvest RC. (a) Scalability of 3M™ Harvest RC across different scales assessed through pressure profile. (b) Turbidity reduction of 3M™ Harvest RC versus depth filtration at different scales

Currently the most widely applied single‐use clarification technology is depth filtration. However, conventional technologies such as depth or membrane filters can foul and cake with higher biomass leading to lower throughputs. The higher contaminant profile can also make depth filter performance more sensitive to batch variations due to naturally derived component variations in depth filters.^8^ Because of these effects, some report up to a 50% safety margin can be deployed during scaling of depth filtration clarification systems from laboratory trials to clinical and commercial deployment.^28^ Because the 3M™ Harvest RC technology is fully defined and utilizes AEX chromatography rather than a combination of surface caking and bed loading, the scaling across different cell culture volumes is much more predictable. To confirm this, we evaluated the performance using a high cell density culture (mAb 3) across three different scales. The differential pressure during filtration followed the same pressure profile across all three scales of bench, pilot, and production scale devices (Figure 4). A standard parameter for assessment of clarification efficiency is turbidity. The clarified filtrate by 3M™ Harvest RC resulted in turbidity values between 3.38 and 3.76 NTU for all three scales. For comparison, the same high cell density culture clarified using the two available scales of the 3M™ Zeta Plus depth filter resulted in a broader and higher range of clarified turbidity between 4.60 and 5.77 NTU. We can conclude that the chromatographic clarification using the 3M™ Harvest RC resulted in a consistently lower turbidity of CCCF compared to a traditional depth filtration approach.

After establishing the chromatographic ability to clarify CCF using the 3M™ Harvest RC, we compared the performance of the 3M™ Harvest RC with other single use clarification technologies with a different cell culture (mAb 2). Three process trains were studied and used to represent the evolution of primarily cell harvest with different single‐use technologies: traditional cell culture clarification using depth filtration (3M™ Zeta Plus 60SP02A), modern chromatographic clarification approach using depth filtration and chromatographic clarification (3M™ Zeta Plus 60SP02A + 3M™ Emphaze™ AEX Hybrid Purifier), and next generation chromatographic clarification with a single stage chromatographic solution (3M™ Harvest RC). The physical characteristics of the filters used in this study are provided in Table 4.

**TABLE 4:** Adsorptive depth filters and hybrid filters' characteristics used in study

Filter	Ligand	Layer configuration	Pore size range (μm)	Typical charge capacity (mg/cm^2^)	Materials of construction
Zeta Plus 60SP02A	Quaternary amine	Dual Layer	0.2–10	0.20 ± 0.02	Cellulose + filter aid + resin binder
Emphaze™ AEX Hybrid Purifier	Quaternary amine	Multilayer	<0.1–0.8^a^	44.0 ± 1.4	Functionalized PP nonwoven + polyamide membrane
3M™ Harvest RC	Quaternary amine	Multilayer	<0.1–10+^a^	22	Functionalized PP nonwoven + PES membrane

^a^ Fibrous media does not have defined pores. We present the “apparent” pore size with respect to contaminant reduction ability.

### 3.2 Depth filter clarification

**FIGURE 5:**
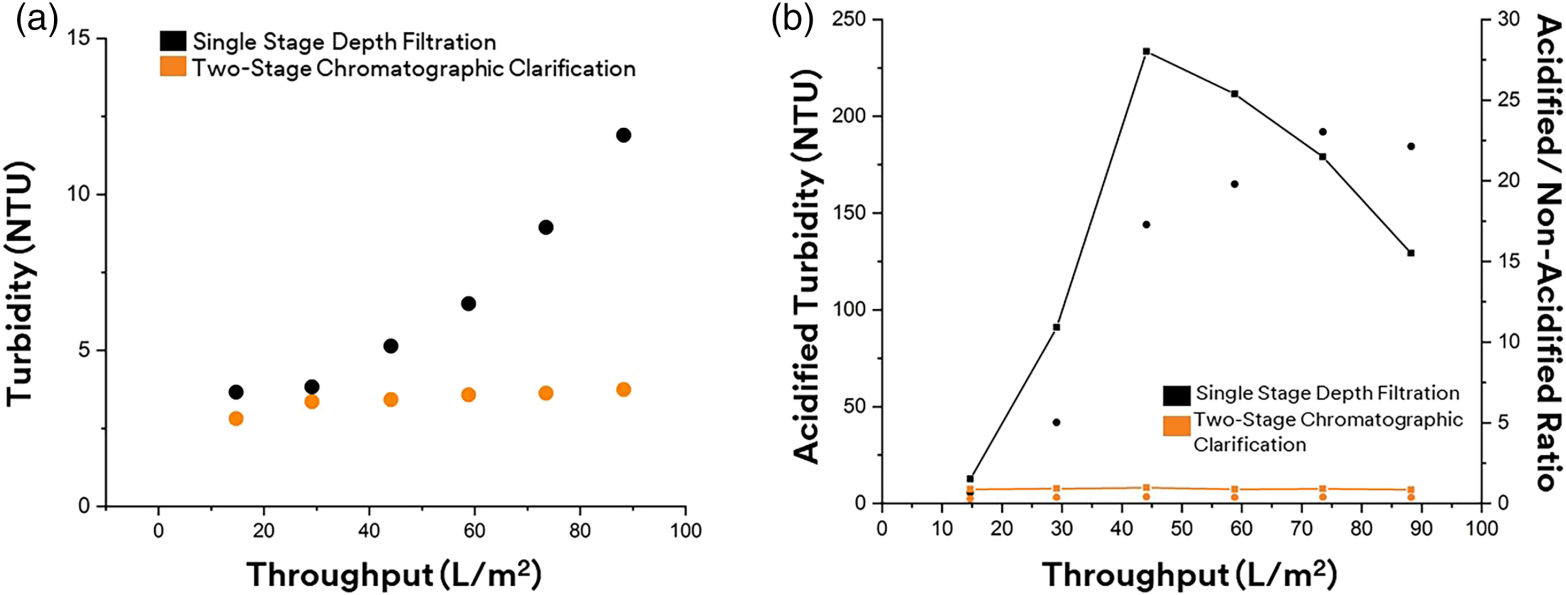
Turbidity of depth filtration and chromatographic clarification. (a) Turbidity after clarification. (b) Acidified turbidity after clarification (circle) and acidified/nonacidified turbidity ratio (solid line)

A single stage depth filter was selected as the base line performance for its prevalence and simplicity. A 3M™ Zeta Plus™ 60SP02A depth filter was chosen because of its dual layer configuration which allows it to capture large particles such as cells as well as smaller particles such as cell debris due to the nominal pore size rating between 0.2 and 10 um. Per the manufacturer's recommendation of usage, a pressure criterion of 15 psid was used as the endpoint for the depth filtration. Using mAb 2 in Table 3, a throughput capacity of 88 L/m^2^ was achieved after reaching the 15 psid. The build‐up in pressure suggests the gradual plugging of pores within the depth filter media. Depth filtration using the 3M Zeta Plus 60SP02A provided a turbidity reduction from an initial harvest turbidity of 2597–12 NTU at a loading of 88 L/m^2^ (Figure 5a). As shown from Figure 5b, removal of soluble contaminants was observed in the first 15 L/m^2^ fraction as illustrated by an acidified turbidity of 5 NTU. It has been reported that acidified turbidity can be used to measure relative abundance of soluble contaminants by lowering the pH of the clarified filtrate or cell culture solution.^29^

As HC‐DNA and high‐molecular weight contaminant begins to breakthrough, precipitation will occur upon acidification. This was observed in subsequent fractions of the single stage depth filtration train when the acidified turbidity increased to ~200 NTU by 88 L/m^2^ (Figure 5b). This suggests the depletion of the anionic charge in the depth filter media over the course of filtration. Despite the rise in acidified turbidity, the turbidity remained low throughout filtration suggesting the ability to still remove large particles contributing to high turbidity but the anionic charge capacity responsible for capture of soluble contaminants was exhausted.

### 3.3 Chromatographic clarification with 3M Emphaze AEX hybrid purifier

The second train exploring the current chromatographic clarification approach used existing technology employing Q‐functionalized nonwoven fibers in the 3M Emphaze™ AEX Hybrid Purifier combined with an initial clarification stage. By selecting the same grade of depth filter (60SP02A) as the previous train, the resulting filtrate can be interpreted as the effect of adding 3M Emphaze™ AEX Hybrid Purifier to achieve chromatographic clarification. As expected, the 60SP02A in this train also reached 15 psid at the same throughput of 88 L/m^2^ as previously observed. The turbidity was initially reduced to approximately 2 NTU by 15 L/m^2^ and never exceeded 4 NTU up till 88 L/m^2^ of loading (Figure 5a). A consistent and stable low turbidity was observed with chromatographic clarification compared to the steady increase in the depth filtration train (Figure 5b). The acidified turbidity also remained below 4 NTU throughout the filtration revealing an acidified/non‐acidified turbidity ratio to be below one throughout the clarification process.

### 3.4 Improved clarified fluid quality using chromatographic clarification

**FIGURE 6:**
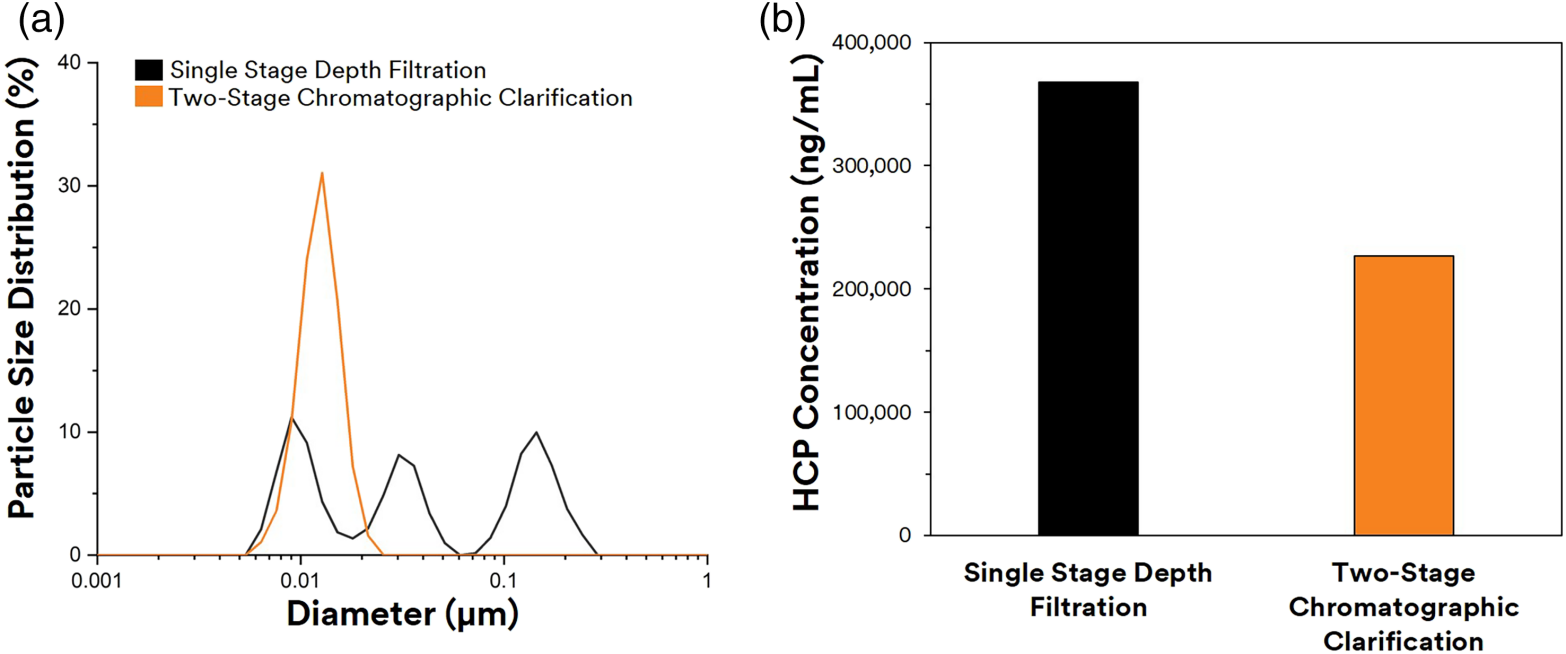
Clarified filtrate Quality. (a) Particle size distribution for depth filtration (orange) and chromatographic clarification (black). (b) Host cell proteins (HCP) concentration for clarified filtrate

In the first clarification train, two peaks were observed centered around 0.01–1 μm suggesting the presence of DNA, chromatin, and cell debris that were not captured during depth filter clarification (Figure 6a). As a result, with the chromatographic clarification method, a monodisperse particle size distribution was observed around 0.01 μm corresponding to the mAb where the peaks corresponding to the presence of DNA, chromatin, and cell debris were not found. This suggests that the highly anionic charge in the Emphaze™ AEX Hybrid Purifier was able to capture many of the soluble contaminants resulting in a lower turbidity and acidified turbidity.

The measured levels of HCPs and acidified turbidity confirmed the DLS findings that chromatographic clarification could remove certain soluble contaminants. As expected, the highest level of HCPs was observed in the conventional depth filtered filtrate at 367,950 ng/ml (Figure 6b). With a multi‐stage clarification train employing a depth filter and 3 M Emphaze AEX Hybrid Purifier, a significantly lower level of HCPs at 226,639 ng/ml was observed (Figure 6b). In the previous sections, we reported before and after acidification turbidity ratio significantly above one for the depth filter filtrate. In contrast, when chromatographic clarification with Emphaze was deployed, the turbidity ratio was below one. This suggests that the inclusion of Emphaze™ can remove HC‐DNA and high molecular weight contaminant which typically would precipitate upon acidification resulting in an increased turbidity. Based on the previous work of Koehler et. al, we estimate that the final genomic DNA concentration in the CCCF <500 ppb for the chromatographic clarified filtrate.^29^

### 3.5 Single step chromatographic clarification with 3M™ Harvest RC

Currently, multiple stages of clarification are needed for chromatographic clarification as demonstrated with the second clarification train. An initial clarification stage focused on the removal of large insoluble debris is required followed by another stage with highly charged media in the Emphaze AEX Hybrid Purifier. The benefits and impact on CCCF quality of chromatographic clarification have been illustrated in previous sections and other reported literature. However, the need to have multiple stages for clarification and chromatographic clarification can add extra complexity, costs, consumables, labor, and logistics.

**FIGURE 7:**
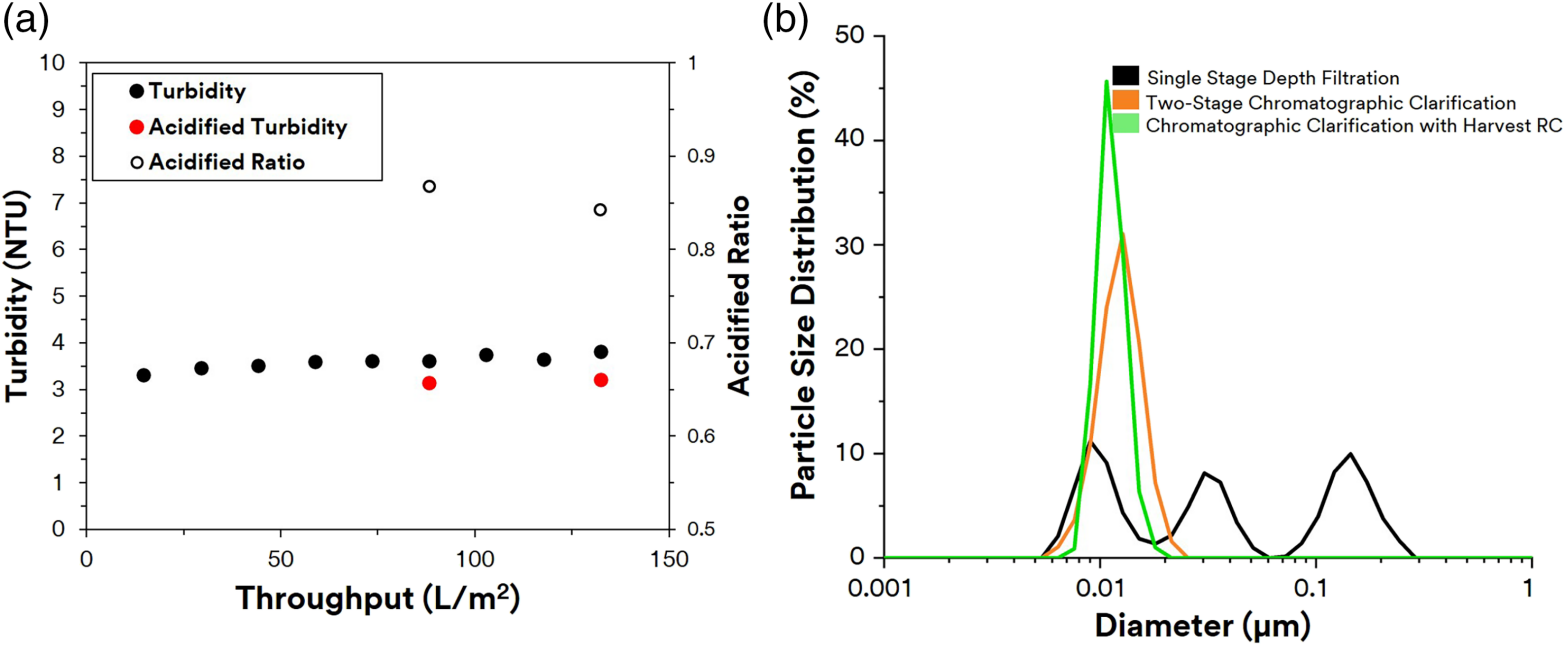
Chromatographic clarification with 3 M Harvest RC. (a) Turbidity and turbidity ratio. (b) Particle size distribution of clarified filtrate

In the last clarification train, we evaluated the performance of performing a single chromatographic clarification stage with the same challenge (mAb 2) compared to current clarification strategies as described in the first two trains. Similar to Emphaze™, 3M™ Harvest RC relies on a quaternary ammonium chemistry for its positive charge on its respective functionalized nonwoven fibers. Whereas 3M Emphaze AEX Hybrid Purifier was designed as the second stage of clarification with a focus on small insoluble and soluble impurities, the charge density and physical attributes of the 3M Harvest RC media have been designed to capture both large contaminants such as cells and debris as well as smaller soluble impurities. As a result, the 3M Harvest RC was explored as a stand‐alone clarification stage without the need for an initial depth filtration stage. Unlike the Zeta Plus™ depth filter, the Harvest RC operates under a consistently low pressure < 2 psid until 110 L/m^2^. After 110 L/m^2^, the pressure increases exponentially before reaching the terminal differential pressure at 132 L/m^2^. The initial turbidity of 2597 NTU was reduced to <4 NTU providing constant outlet turbidity and pressure till the end (Figure 7a). This is consistent with the fact that chromatographic clarification using nonwoven fibers in a relatively open fibrous scaffold relies on capture by charge rather than by size. Thus, one would expect that the pressure would remain constant until the charge capacity is exhausted.

The removal of DNA‐protein complexes using chromatographic clarification by 3M Harvest RC was supported through multiple measurements of impurity removal, DLS, and acidified turbidity. Figure 7 shows that turbidity ratio was measured to be below one throughout the 3M™ Harvest RC filtration process. No precipitation was observed after acidification consistent with the removal of HC‐DNA and other high molecular weight contaminants using highly charged media. Similar with previous observation in chromatographic clarification, the DLS measurement in the 3M Harvest RC clarified fluid was a monodisperse particle size distribution centered around 0.01 μm consistent with the size of protein‐based species, including mAbs (Figure 7b). The HCP concentration for 3M Harvest RC clarification was slightly less than depth filtration clarification at 360,630 ng/ml compared to 367,950 ng/ml. This is expected as 3M Harvest RC Q functional media was not designed to bind to proteins in cell culture conditions but rather larger particles and certain soluble aggregates. All the analytics revealed a similar phenomenon where the chromatographic media of 3M Harvest RC captured many of the soluble contaminants resulting in a low turbidity and acidified turbidity.

### 3.6 Enhanced Protein A performance with chromatographic clarification

**FIGURE 8:**
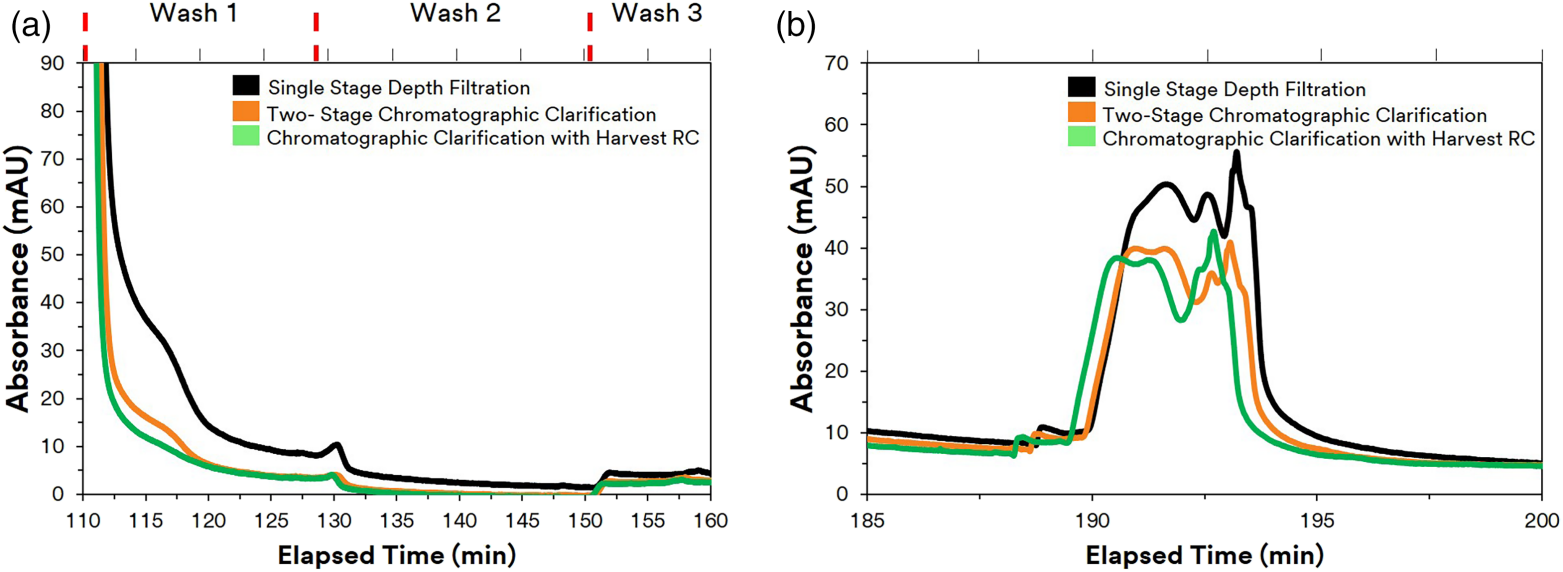
UV absorbance spectra at 280 nm during Protein A chromatography (a) UV absorbance profile during wash steps. (b) Profile during acid stripping for regeneration

We further evaluated the effect of chromatographic clarification on downstream purification steps. Chromatographically clarified filtrate showed improved Protein A performance as revealed during the wash and acid strip steps. Figure 8 illustrates a higher and broader wash peak for material clarified by depth filtration. In the Emphaze AEX Hybrid Purifier clarified material, the Protein A wash peaks were substantially smaller suggesting lower quantities of impurities that were initially bound to the Protein A.

Similarly, with lower HC‐DNA and HCP concentrations from both chromatographic clarification trains, a smaller UV absorbance signal was observed during the Protein A acid stripping step. A peak absorbance of 55 mAU was observed during the acid stripping for material loaded onto the Protein A that had been clarified by depth filtration alone. For the Emphaze clarified filtrate, the peak absorbance was between 45–50 mAU during acid stripping (Figure 8b). Thus, less nonspecific binding of these contaminants was found on the Protein A ligand due to an initial lower HCP and HC‐DNA resulting in a reduced elution of impurities in both the washing and stripping steps of the Protein A column.

**FIGURE 9:**
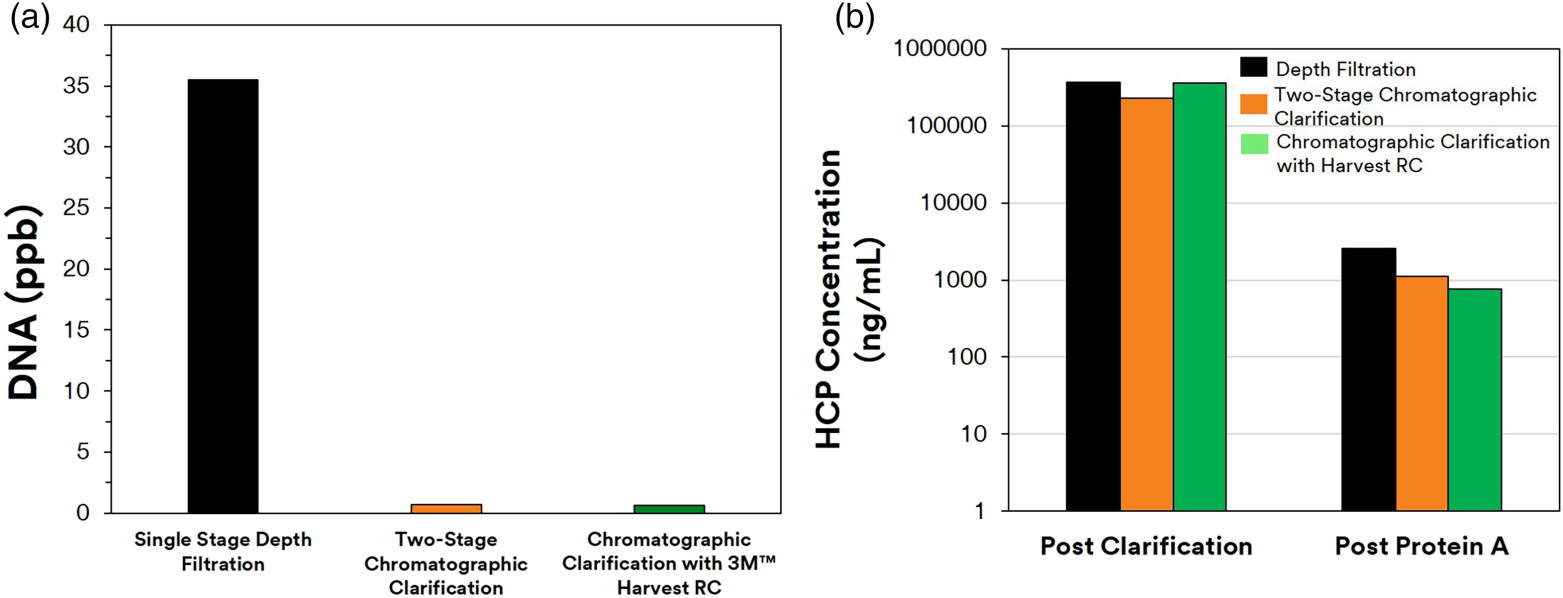
Protein A performance: Reduction in HC‐DNA and host cell proteins (HCP). (a) DNA levels post Protein A. (b) HCP for peer review concentration before and after Protein A

The early removal of soluble contaminants with 3M Emphaze AEX Hybrid Purifier also quantitatively improved HCP and HC‐DNA clearance by Protein A chromatography. The Protein A eluate was found to have 36 ppb HC‐DNA after conventional depth filtration. Chromatographic clarification resulted in >50‐fold improvement in protein A clearance of DNA relative to depth filtration clarification (Figure 9a). In the chromatographic clarification train, the DNA concentration of the protein A eluate was reduced to below the level of detection: from 36 ppb with depth filtration to <0.6 ppb with chromatographic clarification. The reduction of HC‐DNA and HCPs with chromatographic clarification also revealed extra benefit for Protein A HCP clearance. Figure 9b shows that the levels of HCP were reduced from 367,950 before Protein A to 2574 ng/ml after Protein A elution with depth filtered material. The concentration of HCPs was reduced from 226,639 before Protein A to 1124 ng/ml after Protein A with chromatographic clarified filtrate from Emphaze. This is consistent with other literature that reported the ability to use chromatographic clarification to aid in the removal of interfering DNA contaminants to ultimately enable a higher purity in the protein A elution.

The impact of 3 M Harvest RC in clarification to improve downstream purification steps such as Protein A chromatography was also observed. The UV absorbance profiles between the 3 M Harvest RC and Emphaze runs were almost identical to the point of overlapping supporting similar chromatographic performance during clarification (Figure 8). All three wash peaks were lower in the Protein A runs using 3 M Harvest RC compared to conventional depth filter clarification. A similar peak absorbance of 45–50 mAU was also observed during the Protein A acid strip. This indicated that the early removal of contaminants during Harvest RC clarification resulted in better Protein A performance as less contaminants were found bound to the Protein A during the washing and acid stripping steps.

The improved Protein A performance with Harvest RC chromatographic clarification could be observed as the levels of HC‐DNA were also below the level of detection (<0.6 ppb compared to 36 ppb with conventional depth filter clarification). The concentrations of HCPs were reduced from 360,626 ng/ml before Protein A to 754 ng/ml after protein A. In comparison, with conventional depth filter clarification, the concentration of HCPs was reduced from 367,950 ng/ml before protein A to 2574 ng/ml after protein A. Until now, two stages of filtration are needed in order to accomplish chromatographic clarification. To our knowledge, this is the first study to achieve chromatographic clarification in a single stage.

### 3.7 Improved recovery and scalability using Harvest RC

**FIGURE 10:**
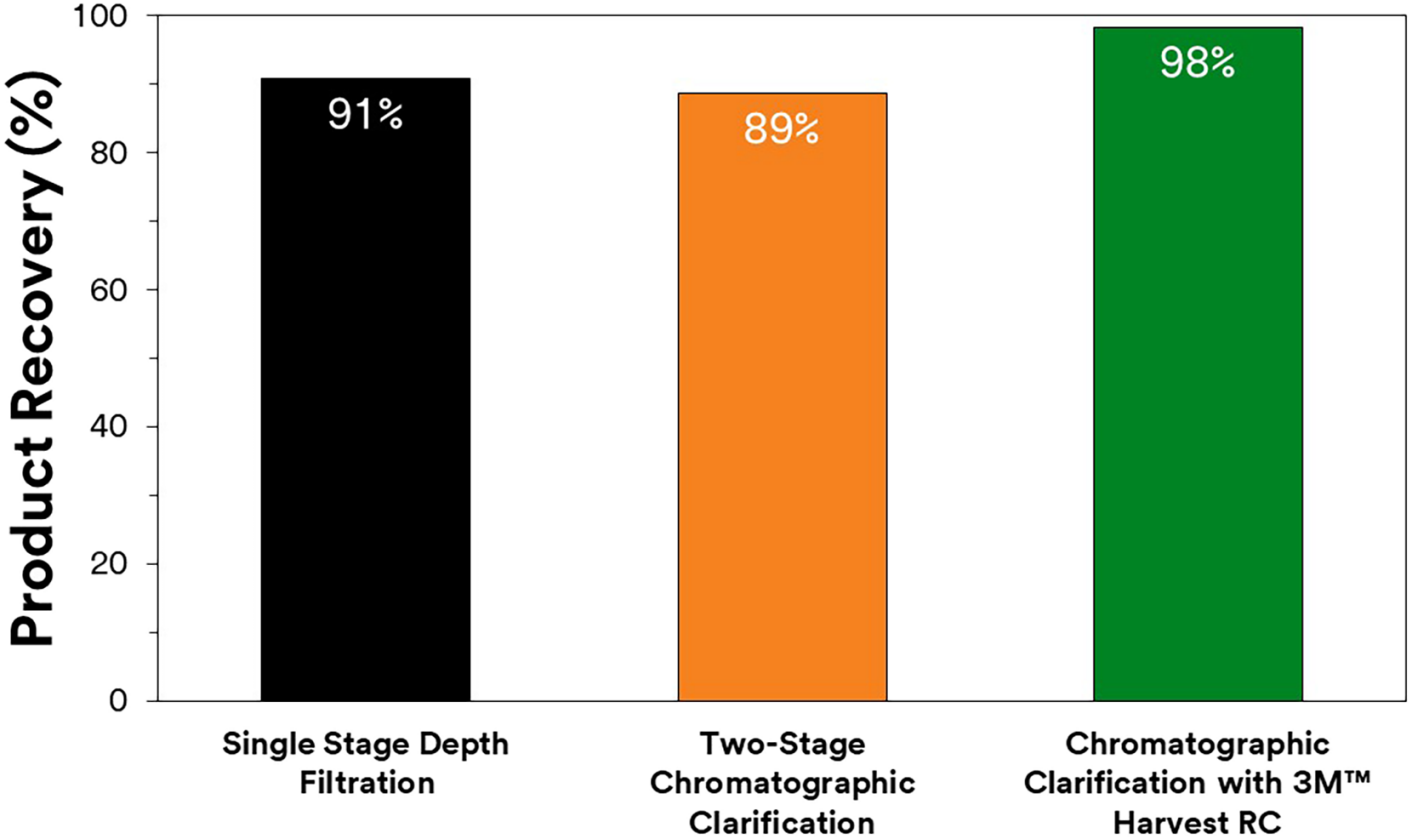
Product recovery after different clarification strategies

Both chromatographic clarified filtrates using 3M Harvest RC or 3M 60SP02A + 3M Emphaze AEX Hybrid Purifier were similar in terms of impurity profile and their impact on Protein A performance. However, the 3M Harvest RC did not require an initial clarification stage. The starting titer of 1.86 g/L was reduced to 1.69 g/L after 3 M Zeta Plus depth filtration. This translates to a 91% product recovery rate after depth filtration consistent with what has been reported in literature. The second train for chromatographic clarification had an overall product recovery rate of 89%. As expected, the product recovery rate is lower for the second train as an extra unit operation was needed to deploy chromatographic clarification. Because the same 3 M Zeta Plus depth filter was used, it can be inferred that a 2% product loss was observed across the 3 M Emphaze AEX Hybrid Purifier. As presented in Figure 10 3M Harvest RC had ~98% product recovery with the same concentration before and after clarification. This higher product recovery has large ramifications in terms of process simplicity and process economics.

### 3.8 Enabling improved economics for clarification through cost analysis

**FIGURE 11:**
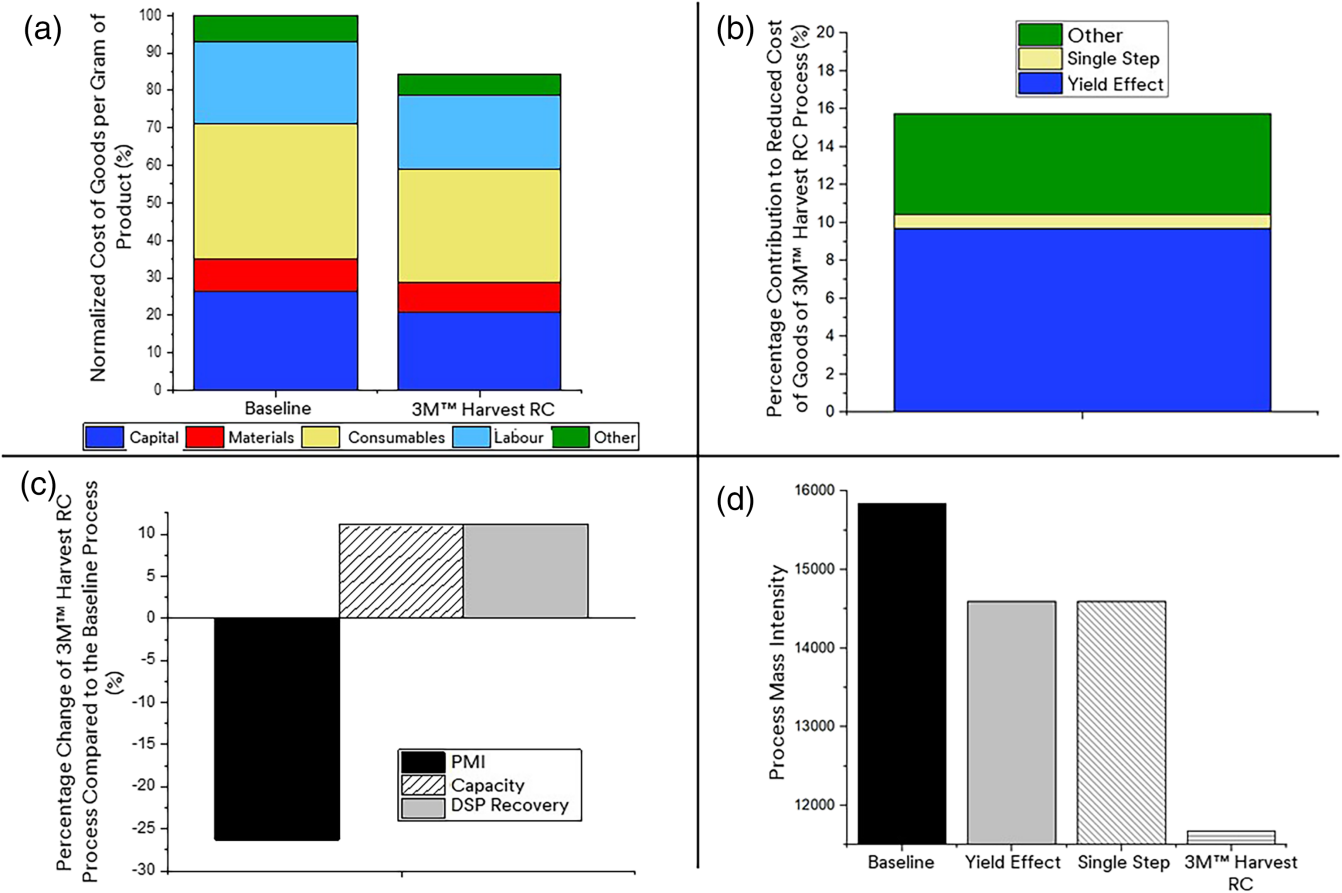
Biosolve model results. (a) shows the percentage contribution of capital (blue), materials (red), consumables (yellow), labour (light blue) and other costs(green) to the CoGs for the baseline process and the 3M ™ Harvest RC process. All values were normalized to the mAb manufacturing cost per gram for the baseline process. (b) shows the relative contribution to the reduction in the mAb manufacturing cost of the increased downstream processing (DSP) yield (blue), combining the two clarification steps into one (yellow), the removal of the peruse flush (red) and other factors (green) such as the reduced number of capsules, reduced waste and differences in capsule cost. The percentages presented are relative to the mAb manufacturing cost for the baseline process. (c) shows impact of implementation of 3M™ Harvest RC on the process mass intensity (black), capacity (white) that is, the quantity of product than can be produced using the process) and the DSP recovery (gray). (d) shows the reduction in the process mass intensity (PMI) due to increased DSP yield (gray), combining the two clarification steps into one (diagonal hash), the removal of the peruse flush (horizontal hatch) and 3M™ Harvest RC (white) compared to the baseline process (black)

In the second half of this study, we explored the financial ramifications of deploying 3 M Harvest RC as a single step chromatographic clarification. A BioSolve™ cost analysis was modeled using the experimental results from previous sections. Figure 11a shows the relative contribution of capital, materials, consumables, labor, and other costs to the total cost of the 3M™ Harvest RC process compare to the baseline process. Overall, mAb manufacturing costs are reduced by approximately 16% for the Harvest RC process. A reduction, in terms of cost per gram of product is seen across all cost categories, with the largest reductions being in capital and other costs. The reduced capital cost is primarily driven by lower requirements for utility generation and storage, buffer storage and filter holders.

The reduction in other costs observed includes insurance; waste management; maintenance and utilities. The reduction in these costs are: 11.6%; 6.6%; 12.1 and 12.5%, respectively. Since all these reductions, except for the waste management costs, are around 11%, they are thought to be driven primarily by the 11% increase in the capacity of the process due to the 11% increase in DSP yield. With respect to the waste management costs, which are reduced by less than 11%, it is thought that the increased yield, as shown from Figure 10, will give rise to more product to be handled by the downstream process which therefore requires more consumables downstream to deal with the increased amount of product. Consequently, some of the reduction in cost, resulting from the increased yield, is balanced out by an increase in the cost of disposing of these extra consumables.

Figure 11b shows the contribution of the various features of Harvest RC to the overall 16% reduction in mAb manufacturing costs. By far the largest contributor, 10 out of the 16%, to the overall reduction in mAb manufacturing costs, is the downstream process yield increase that results from the use of 3M™ Harvest RC and gives rise to an increase in mAb production capacity of approximately 11% (Figure 11c). This is because of the high value of the mAb product when compared to the materials used to produce and purify it and is intuitive (i.e., if the mAb did not have that relatively high value then the process would be economically unviable). Just under 1% point of the reduction in manufacturing costs is due to moving from multiple clarification steps to a single clarification step. The remaining cost savings, labeled as other, covers the reduced number of consumables and differences in consumable cost.

In addition to the cost savings resulting from the use of Harvest RC, a reduction in the process mass intensity (PMI) of ~26% is also observed—as shown in Figure 11c. PMI is a measure of the quantity of process water, materials and consumables required. A lower PMI indicates that less of these things are required to manufacture a gram of product and therefore indicates that the process has a reduced environmental impact. This reduced PMI is observed for a number of reasons which include: a reduction in the quantity of process water required for flushing; lower clarification consumable requirements, that is, 10 capsules used during the Harvest RC clarification compared to 24 capsules for the baseline process as shown in Table 2; and the increased yield which results in a more efficient process overall.

## 4 CONCLUSION

To our knowledge, this is the first study to use chromatography to capture whole cells, cell debris, and soluble impurities in a single clarification step for a variety of cell cultures. Overall, the findings suggest that the 3M™ Harvest RC is well suited for past, current, and upcoming challenging cell cultures. The results indicate the benefits of utilizing chromatography in the form of the 3M™ Harvest RC such as reduced DNA, higher recovery yield, robust scalability, and higher purity compared to traditional clarification technologies. These findings were further supported using an economic model to show that the inclusion of 3M Harvest RC has a beneficial impact in terms of reducing mAb manufacturing cost and reducing environmental impact. These benefits are a strong function of product yield improvement but other factors such as simplified deployment, faster processing and process simplification also play a role as well.

## AUTHOR CONTRIBUTIONS

**Aaron Almeida:** Data curation (lead); formal analysis (lead); investigation (lead); methodology (lead); supervision (lead); writing – original draft (lead); writing – review and editing (lead). **David Chau:** Conceptualization (lead); data curation (lead); formal analysis (lead); investigation (lead); methodology (lead); project administration (lead); supervision (lead); validation (lead); visualization (lead); writing – original draft (lead). **Thomas Coolidge:** Data curation (supporting); formal analysis (supporting); investigation (supporting); methodology (supporting); validation (supporting); visualization (supporting); writing – original draft (supporting); writing – review and editing (supporting). **Hani El‐Sabbahy:** Data curation (supporting); formal analysis (supporting); visualization (supporting); writing – review and editing (supporting). **Kevin Jose:** Data curation (supporting); investigation (supporting). **Masayuki Nakamura:** Data curation (supporting); formal analysis (supporting); investigation (supporting); methodology (supporting); writing – original draft (supporting). **Steven Hager:** Supervision (supporting); writing – review and editing (supporting). **Alexei M Voloshin:** Conceptualization (supporting); formal analysis (supporting); methodology (supporting); supervision (supporting); writing – review and editing (supporting).

### PEER REVIEW

The peer review history for this article is available at https://publons.com/publon/10.1002/btpr.3227.

## Data Availability

The data that support the findings of this study are available from the corresponding author upon reasonable request.

## References

[1] Lu R‐M , Hwang Y‐C , Liu I‐J , et al. Development of therapeutic antibodies for the treatment of diseases. J Biomed Sci. 2020;27(1):1. doi:10.1186/s12929-019-0592-z 31894001 PMC6939334

[2] Walsh G . Biopharmaceutical benchmarks 2010. Nat Biotechnol. 2010;28(9):917‐924. doi:10.1038/nbt0910-917 20829826

[3] Weng Z , Jin J , Shao C , Li H . Reduction of charge variants by CHO cell culture process optimization. Cytotechnology. 2020;72(2):259‐269. doi:10.1007/s10616-020-00375-x 32236800 PMC7192992

[4] Stephenson FH . Calculations for Molecular Biology and Biotechnology. Academic Press; 2016.

[5] Handbook of Methods and Instrumentation in Separation Science. Vol 1. Academic Press; 2009.

[6] Singh N , Pizzelli K , Romero JK , et al. Clarification of recombinant proteins from high cell density mammalian cell culture systems using new improved depth filters. Biotechnol Bioeng. 2013;110(7):1964‐1972. doi:10.1002/bit.24848 23334838

[7] Dryden WA , Larsen LM , Britt DW , Smith MT . Technical and economic considerations of cell culture harvest and clarification technologies. Biochem Eng J. 2021;167:107892. doi:10.1016/j.bej.2020.107892

[8] Singh N , Arunkumar A , Chollangi S , Tan ZG , Borys M , Li ZJ . Clarification technologies for monoclonal antibody manufacturing processes: Current state and future perspectives. Biotechnol Bioeng. 2016;113(4):698‐716. doi:10.1002/bit.25810 26302443

[9] Hong JS , Azer N , Agarabi C , Fratz‐Berilla EJ . Primary clarification of CHO harvested cell culture fluid using an acoustic separator. Jove J Vis Exp. 2020;159:e61161. doi:10.3791/61161 32478745

[10] Shukla AA , Gottschalk U . Single‐use disposable technologies for biopharmaceutical manufacturing. Trends Biotechnol. 2013;31(3):147‐154. doi:10.1016/j.tibtech.2012.10.004 23178074

[11] Frank GT . Transformation of biomanufacturing by single‐use systems and technology. Curr Opin Chem Eng. 2018;22:62‐70. doi:10.1016/j.coche.2018.09.006

[12] Khanal O , Singh N , Traylor SJ , et al. Contributions of depth filter components to protein adsorption in bioprocessing. Biotechnol Bioeng. 2018;115(8):1938‐1948. doi:10.1002/bit.26707 29663326

[13] Yigzaw Y , Piper R , Tran M , Shukla A . Exploitation of the adsorptive properties of depth filters for host cell protein removal during monoclonal antibody purification. Biotechnol Prog. 2006;22:288‐296. doi:10.1021/bp050274w 16454522

[14] Fouling of an Anion Exchange Chromatography Operation in a Monoclonal Antibody Process: Visualization and Kinetic Studies ‐ Close ‐ 2013 ‐ Biotechnology and Bioengineering. Wiley Online Library; 2021. doi:10.1002/bit.24898 PMC3840701 23483524

[15] Jin Z . Expanded bed adsorption – challenges and advances in column and process design. Prod Dev. 2015;35:66‐78.

[16] May T , Pohlmeyer K . Improving Process Economy with Expanded‐Bed Adsorption Technology. BioProcess International. 2011;9:32‐36.

[17] Shabram P , Vellekamp G , Ruan Q , Scandella C . Purification of adenovirus. In: Curiel DT , ed. Adenoviral Vectors for Gene Therapy (Second Edition). Academic Press; 2016:197‐230. doi:10.1016/B978-0-12-800276-6.00008-5

[18] Improved HCP Reduction Using a New, all‐Synthetic Depth Filtration Media within an Antibody Purification Process. Biotechnology Journal ‐ Wiley Online Library; 2019. doi:10.1002/biot.201700771 29710434

[19] Development of adsorptive hybrid filters to enable two‐step purification of biologics. 2021. doi:10.1080/19420862.2016.1267091 PMC5297532 27929735

[20] Evaluating Adsorptive Filtration As a Unit Operation for Virus Removal. BioProcess International. 2015;13(2):36‐44.

[21] Castro‐Forero A , Jokondo Z , Voloshin A . Anion‐Exchange Chromatographic Clarification. BioProcess International. 2015;13:52‐57.

[22] Voloshin A , Smirnov D , Wessel W , Collins I , Hager S . Enabling Higher Post Protein A Product Purity Using Novel Chromatographic Clarification Approach. La Vague. 2016;2(5):480‐499. doi:10.4161%2Fmabs.2.5.12645

[23] Iskra T , Bolton GR , Coffman JL , Godavarti R . The effect of protein a cycle number on the performance and lifetime of an anion exchange polishing step. Biotechnol Bioeng. 2013;110(4):1142‐1152. doi:10.1002/bit.24781 23138874

[24] Kang Y , Hamzik J , Felo M , et al. Development of a novel and efficient cell culture flocculation process using a stimulus responsive polymer to streamline antibody purification processes. Biotechnol Bioeng. 2013;110(11):2928‐2937. doi:10.1002/bit.24969 23740533 PMC3812681

[25] Kravitz R , Vredenburgh W , Chrostowski V , Bleck G . Achieving unique synergies in antibody expression. Genet Eng Biotechnol News. 2019;39(7):55‐57. doi:10.1089/gen.39.07.16

[26] Pieracci JP , Armando JW , Westoby M , Thommes J . Industry review of cell separation and product harvesting methods. In: Jagschies G , Lindskog E , Łącki K , Galliher P , eds. Biopharmaceutical Processing. Elsevier; 2018:165‐206. doi:10.1016/B978-0-08-100623-8.00009-8

[27] Goldrick S , Joseph A , Mollet M , et al. Predicting performance of constant flow depth filtration using constant pressure filtration data. J Membr Sci. 2017;531:138‐147. doi:10.1016/j.memsci.2017.03.002

[28] Kandula S , Babu S , Jin M , Shukla AA . Design of a filter train for precipitate removal in monoclonal antibody downstream processing. Biotechnol Appl Biochem. 2009;54(3):149‐155. doi:10.1042/BA20090181 19656082

[29] Koehler KC , Jokondo Z , Narayan J , Voloshin AM , Castro‐Forero AA . Enhancing Protein A performance in mAb processing: A method to reduce and rapidly evaluate host cell DNA levels during primary clarification. Biotechnol Prog. 2019;35(6). doi:10.1002/btpr.2882 PMC7003430 31276322

